# Can baseline serum microRNAs predict response to TNF-alpha inhibitors in rheumatoid arthritis?

**DOI:** 10.1186/s13075-016-1085-z

**Published:** 2016-08-24

**Authors:** Bart V. J. Cuppen, Marzia Rossato, Ruth D. E. Fritsch-Stork, Arno N. Concepcion, Yolande Schenk, Johannes W. J. Bijlsma, Timothy R. D. J. Radstake, Floris P. J. G. Lafeber, on behalf of all SRU investigators

**Affiliations:** 1 Rheumatology and Clinical Immunology, University Medical Center Utrecht, Heidelberglaan 100, 3584 CX Utrecht, The Netherlands; 2 Laboratory of Translational Immunology, University Medical Center Utrecht, Heidelberglaan 100, 3584 CX Utrecht, The Netherlands; 3 1st Medical Department & Ludwig Boltzmann Institute of Osteology at the Hanusch Hospital of WGKK and AUVA Trauma Centre Meidling, Hanusch Hospital, Heinrich-Collin-Straße 30, 1140 Vienna, Austria; 4 Sigmund Freud University, Freudplatz 1, 1020 Vienna, Austria; 5 Rheumatology, Diakonessen Hospital, Bosboomstraat 1, 3582 KE Utrecht, The Netherlands

**Keywords:** miRNA, Rheumatoid arthritis, Prediction, Prognosis, Response, TNF-alpha inhibitor

## Abstract

**Background:**

In rheumatoid arthritis, prediction of response to TNF-alpha inhibitor (TNFi) treatment would be of clinical value. This study aims to discover miRNAs that predict response and aims to replicate results of two previous studies addressing this topic.

**Methods:**

From the observational BiOCURA cohort, 40 adalimumab- (ADA) and 40 etanercept- (ETN) treated patients were selected to enter the discovery cohort and baseline serum profiling on 758 miRNAs was performed. The added value of univariately selected miRNAs (*p* < 0.05) over clinical parameters in prediction of response was determined by means of the area under the receiver operating characteristic curve (AUC-ROC). Validation was performed by TaqMan single qPCR assays in 40 new patients.

**Results:**

Expression of miR-99a and miR-143 predicted response to ADA, and miR-23a and miR-197 predicted response to ETN. The addition of miRNAs increased the AUC-ROC of a model containing only clinical parameters for ADA (0.75 to 0.97) and ETN (0.68 to 0.78). In validation, none of the selected miRNAs significantly predicted response. miR-23a was the only overlapping miRNA compared to the two previous studies, however inversely related with response in one of these studies. The reasons for the inability to replicate previously proposed miRNAs predicting response to TNFi and replicate those from the discovery cohort were investigated and discussed.

**Conclusions:**

To date, no miRNA consistently predicting response to TNFi therapy in RA has been identified. Future studies on this topic should meet a minimum of standards in design that are addressed in this study, in order to increase the reproducibility.

**Electronic supplementary material:**

The online version of this article (doi:10.1186/s13075-016-1085-z) contains supplementary material, which is available to authorized users.

## Background

Rheumatoid arthritis (RA) is a chronic, disabling disease that mainly affects the synovial joints, with a prevalence of 0.5–1.0 % in Western countries [1, 2]. The introduction of TNF-α-inhibiting therapy (TNFi), such as adalimumab (ADA) and etanercept (ETN), has dramatically improved the outlook for RA patients. Nevertheless, a substantial proportion of patients (approximately 30–40 %) fail to respond to TNFi therapy [3, 4]. As we cannot predict before initiation of therapy which patients will be non-responders [5], TNFi treatment is administered in a trial and error approach. However in the time frame from initiation of therapy until response can be judged, which is usually 3–6 months later, non-responding patients suffer from uncontrolled disease with possible joint damage and the potential harmful side effects from treatment. The challenge is therefore to identify responders and non-responders to TNFi beforehand, so that TNFi use or considering alternatives can be encouraged.

microRNAs (miRNAs) are a large family of highly conserved noncoding genes that play a fundamental role in biological processes by controlling protein expression [6–8]. miRNAs execute these actions by binding to protein-coding messenger RNAs (mRNAs), resulting in translational repression or mRNA degradation [7]. Besides intracellulary, miRNAs are also found in several biological fluids, including saliva, plasma, serum and urine, either circulating in conjunction with specific carrier proteins or enclosed in extracellular vesicles [9, 10]. Exploring the use of circulating miRNAs as biomarkers for diseases has gained momentum in recent years because of the easy accessibility, the associations with specific disease conditions and their good stability [10, 11]. In RA, a systemic inflammatory disease primarily manifesting in the joints, biomarkers in the circulation would intuitively not be the most relevant compartment. However, the levels of miRNAs are frequently higher in the circulation than in the synovial fluid and correlate with disease activity in RA, indicating that the systemic compartment provides a useful compartment for studying the ongoing pathophysiological processes [12] In addition, abnormal expression of both synovial and systemic miRNAs have been linked to disease activity and pathogenesis, even though their direct targets are not always known [13–19]. Three recent studies focused on the prediction of response to therapy in RA by using circulating miRNAs, of which two investigated response to therapy with TNFi [20, 21] and one to rituximab [22]. According to these studies, promising predictors for TNFi therapy were miR-22 [20], miR-23a [21], miR-223 [21], and miR-886 [20]. Circulating miR-23a seems of particular interest, since it was the only identified candidate biomarker that was overlapping among both studies in univariate analyses. However, upregulation of miR23a was found in whole blood [20], whereas a downregulation was found in serum [21] of future responders.

In this study we explored the serum miRNAs associated with good and bad response to TNFi therapy, in order to replicate the results that have been published before. In addition to the previous studies performed, we involved clinical parameters in the prediction and attempted to validate the miRNAs and prediction models in a separate cohort.

## Methods

### Clinical data collection

Patients initiating ADA or ETN therapy were selected from the “Biologicals and Outcome Compared and predicted Utrecht region in Rheumatoid Arthritis” (BiOCURA) study. BiOCURA is an observational cohort, in which RA patients eligible for biological treatment according to regular clinical practice were enrolled and followed after start of treatment, in one academic hospital and seven regional hospitals in the Netherlands (see Acknowledgements). Re-inclusion after switching to a different biological treatment was possible, at which patients re-entered baseline again. The study was approved by the local ethics committee of the University Medical Center Utrecht and the institutional review boards of the participating centers, and was performed in accordance with the Declaration of Helsinki. Informed consent was obtained from each patient.

Trained nurses gathered all data during a dedicated visit, which included all clinical parameters, joint counts and collection of blood. Visits were scheduled at baseline (before initiation) and after 3, 6, and 12 months of treatment. Disease activity was assessed using the disease activity score based on a 28-joint count (DAS28) [23] and subsequently the European League Against Rheumatism (EULAR) response was calculated [24]. This study design, allowed the determination of a clinical response of each patient, on the basis of three EULAR responses over the course of 1 year.

### Patient selection

Two separate cohorts were composed from the available patients in the BiOCURA study: a discovery cohort was used to screen the expression of a full panel of 758 miRNAs, while a validation cohort was used to test if the results found in the discovery phase were reproducible. The discovery cohort was formed by selecting the most extreme patients regarding clinical response, among all ADA- and ETN-treated patients included between June 2009 (start of BiOCURA) and October 2012 (*n* = 74 ADA and *n* = 68 ETN). The top responding patients (*n* = 20 for both ADA and ETN, from now on called “responders”), were identified by the selection of patients with the best three EULAR responses over the course of 1 year. The selection of bad responders (*n* = 20 for both ADA and ETN, from now on called “non-responders”), was based on the most negative EULAR responses over the course of 1 year and/or (early) discontinuation of TNFi treatment due to inefficacy. Patients with a baseline DAS28 < 2.6 were excluded from the analysis in order to reduce the chance that limited improvement in DAS28 resulted in misclassification as EULAR non-responders. For validation of results, responders (*n* = 10 for both ADA and ETN) and non-responders (*n* = 10 for both ADA and ETN) were selected using the same criteria as in the discovery cohort, among patients included from October 2012 until June 2015 (*n* = 25 ADA and *n* = 40 ETN). Since the validation cohort was smaller, relatively more patients were selected and the differences in clinical outcome between responders and non-responders were less extreme. The baseline characteristics for responders and non-responders are shown in Table 1 and for responders and non-responders split per cohort in Additional file 1. Additionally, the baseline characteristics for the discovery and validation cohorts are shown in Additional file 2.

**Table 1:** Baseline characteristics of responders and non-responders, split for treatment received

Item	ADA (*n* = 60)			ETN (*n* = 60)		
	Non-resp	Resp	*p* value	Non-resp	Resp	*p* value
	(*n* = 30)	(*n* = 30)		(*n* = 30)	(*n* = 30)	
Female gender, n (%)	21 (70)	21 (70)	1.00	25 (83)	21 (70)	0.36
Age, mean years ± sd	54.4 ± 10.9	53.5 ± 12.7	0.76	58.3 ± 9.2	55.1 ± 10.5	0.22
Current smoker, n (%)	16 (53)	8 (27)	0.06	8 (27)	7 (23)	1.00
RF positivity, n (%)	16 (53)	21 (70)	0.29	20 (67)	22 (73)	0.78
ACPA positivity, n (%)	19 (63)	19 (63)	1.00	19 (63)	26 (87)	0.07
CRP, mg/l median (IQR)	5.2 (1.6–10.5)	5.5 (2.0–12.3)	0.78	4.0 (2.0–9.0)	8.5 (4.0–18.3)	**0.03**
No. of previously used bDMARDs						1.00
0, n (%)	20 (67)	23 (78)		22 (73)	22 (73)	
1, n (%)	9 (30)	7 (23)		7 (23)	7 (23)	
2, n (%)	1 (3)	0 (0)		1 (3)	1 (3)	
Concomitant treatment, n (%)	29 (97)	29 (97)	1.00	27 (90)	29 (97)	0.61
MTX, n (%)	21 (70)	27 (90)	0.10	18 (60)	25 (83)	0.08
SSZ, n (%)	2 (7)	4 (13)	0.67	4 (13)	2 (7)	0.67
HCQ, n (%)	8 (27)	7 (23)	1.00	10 (33)	11 (37)	1.00
GC, n (%)	15 (50)	4 (13)	**0.01**	11 (37)	6 (20)	0.25
Baseline DAS28, mean ± sd	3.9 ± 1.4	4.7 ± 0.9	**0.01**	4.3 ± 1.2	4.6 ± 0.9	0.21
TJC, median (IQR)	5.0 (1.0–13.0)	7.0 (4.0–14.3)	0.35	6.5 (2.8–11.3)	5.0 (2.8–11.3)	0.87
SJC, median (IQR)	0.0 (0.0–4.0)	2.0 (0.0–4.0)	**0.03**	1.0 (0.0–3.3)	2.0 (0.8–4.0)	0.20
VAS-GH, mean ± sd	55.2 ± 23.8	63.8 ± 22.0	0.15	55.5 ± 22.8	55.1 ± 10.5	0.76
ESR, median mm/hr (IQR)	11.0 (3.8–26.0)	16.5 (9.0–32.0)	0.14	13.0 (5.8–33.8)	21.0 (14.3–39.5)	0.07

RA patients were selected from the observational BiOCURA cohort based on treatment outcome over the course of 1 year after the start of treatment with either ADA or ETN. The presented clinical characteristics for responders and non-responders refer to the values present before treatment initiation. *P* values of comparisons between responders and non-responders were calculated by means of an independent sample *t* test, Mann-Whitney *U* test, Fisher’s exact test (2*2) or chi-square (>2*2) based on the distribution of the clinical parameter. Bold *p* values indicate significant associations (*p* < 0.05)

*ACPA* anti-citrullinated protein antibody, *ADA* adalimumab, *bDMARDs* biological disease-modifying antirheumatic drugs, *CRP* C-reactive protein, *ESR* erythrocyte sedimentation rate, *ETN* etanercept, *GC* glucocorticoid, *HCQ* hydroxychloroquine, *IQR* interquartile range, *MTX* methotrexate, *RF* rheumatoid factor, *SJC* swollen joint count, *SSZ* sulfasalazine, *TJC* tender joint count, *VAS-GH* visual analogue scale of general health

### miRNA analyses

#### Blood processing and RNA extraction

Blood was collected in Vacutainer® SSTII tubes (BD, Franklin Lakes, NJ, USA) and processed immediately after clotting. Samples were centrifuged for 10 min at 1500 g at room temperature and serum was aliquoted and stored at −80 °C until use. RNA was extracted from 240 μl of serum using the miRcury RNA Isolation kit for Biofluids (Exiqon), according to the manufacturer’s instructions. During extraction, 300 pg of a synthetic miRNA (Arabidopsis thaliana ath-miR-159a) was added to each sample as a spike-in to monitor technical variability along the isolation procedure and for later normalization.

#### miRNA profiling

miRNA profiling was performed by TaqMan RT-qPCR on the OpenArray platform (Life Technologies, Carlsbad, CA, USA). This method allows the simultaneous analysis of 758 miRNAs, split into two equal pools (A and B). Manufacturer’s instructions were followed with minor adjustments. Briefly, 2.5ul of isolated serum RNA was reverse-transcribed by using the miRNA multiplex RT primers pools, either v2.1 for pool A or v3.0 for pool B, and the TaqMan miRNA reverse transcription kit (Life Technologies). RT products were pre-amplified using the Megaplex PreAmp Primers pools A and B in the presence of the TaqMan PreAmp Master Mix (Life Technologies), by using the following thermal cycler conditions: 10 min, 95 °C; 2 min, 55 °C; 2 min, 72 °C and 16 cycles of 15 sec, 95 °C and 4 min, 60 °C and one single cycle of 10 min, 96 °C. The miRNA OpenArray profiling was performed on the amplified cDNA, diluted to 1:40, with 0.1 × TE buffer pH 8.0 and subsequently 1:2 by using the TaqMan OpenArray Master Mix on the QuantStudio 12 K Flex Real-Time PCR System (Life Technologies).

miRNA profiling data was analyzed using the Relative Quantification application in the online accessible Thermo Fisher Cloud (https://apps.thermofisher.com/apps/dashboard/), using the relative threshold cycle (Crt) and the comparative threshold cycle method [25]. Briefly, miRNA expression was calculated after normalization by exogenous ath-miR-159a spike-in (ΔCrt = Crt mean target – Crt mean miR-159a). The relative fold change (FC) of each sample was determined by setting the FC of a random ADA or ETN non-responder sample at 1, and calculating the FC compared to this reference (FC = 2^–ΔΔCrt^, where –ΔΔCrt = ΔCrt reference – ΔCrt sample). Low expressed miRNAs, i.e., having Crt higher than 27 were set to 27, and samples with a low amplification quality (i.e., amplification score < 1.24) were excluded from the analysis.

#### Individual miRNA analysis

miRNA-specific TaqMan Real-Time quantitative PCR (RT-qPCR) assays were purchased from Life Technologies for hsa-miR-23a-3p (ID 000399), hsa-miR-99a-5p (ID 000435), hsa-miR-143-3p (ID 002249), hsa-miR-197-3p (ID 000497), and for the exogenous control ath-miR-159a (ID 000338). From 2.5 μl baseline serum RNA, cDNA was synthesized by using individual miRNA-specific RT primers contained in the miRNA assay in the presence of 3.3 U/μl MultiScribe RT enzyme (Life Technologies), by using the following thermal cycler conditions: 10 min, 4 °C; 30 min,16 °C; 30 min, 42 °C; and 5 min, 85 °C. Circulating miRNA levels were quantified in duplicate from 3 μl cDNA, with TaqMan Fast Advance Master Mix and specific primers of the miRNA assay, using the following amplification condition on the Quantstudio 12 K Flex Real-Time PCR system: 2 min, 50 °C; 20 sec, 95 °C; 40 cycles of 1 sec, 95 °C; and 20 sec, 60 °C. RT-qPCR data were calculated as described above, with the difference that baseline threshold cycles (Ct) were used.

### Statistical analyses

Differential expression of miRNAs between responders and non-responders was calculated separately for ADA and ETN by means of an independent sample *t* tests on the –ΔΔCrt/–ΔΔCt, with a threshold for significance of 0.05 (uncorrected *p* value). The levels of differentially expressed miRNAs were plotted in GraphPad Prism (GraphPad, La Jolla, CA, USA) as FC of responders *versus* non-responders. Validation was considered successful when both the *t* test was significant and plots of the FC showed the same direction (i.e., up/downregulation).

In order to determine the added value of the miRNAs over clinical parameters, we built two prediction models for each treatment, using multivariable logistic regression. The first model consisted of all baseline clinical parameters that were significantly different between responders and non-responders (the “clinical model”). The second model included the clinical parameters and the selected miRNAs (–ΔΔCrt values) (the “combined model”). Per model, the area under the receiver operating characteristic curve (AUC-ROC) was calculated as an indicator of the predictive ability. We considered an AUC-ROC of < 0.7 limitedly, 0.7–0.8 moderately and > 0.8 highly predictive of response. The sensitivity and specificity were calculated for the best cutoff value per model, according to Youden’s index [26]. Evaluation of the added value of miRNAs was based on the increase of predictive abilities when switching from the clinical to the combined model. In order to validate the findings from multivariable analysis, the prediction rules of the clinical and combined models were applied in the validation cohort, thereby freezing the regression coefficients of the individual parameters from the original model. Again, the AUC-ROC, the sensitivity and specificity were calculated to interpret the added value of miRNAs over clinical parameters alone.

## Results

### Identification of miRNAs as predictor of TNFi response

**Fig. 1:**
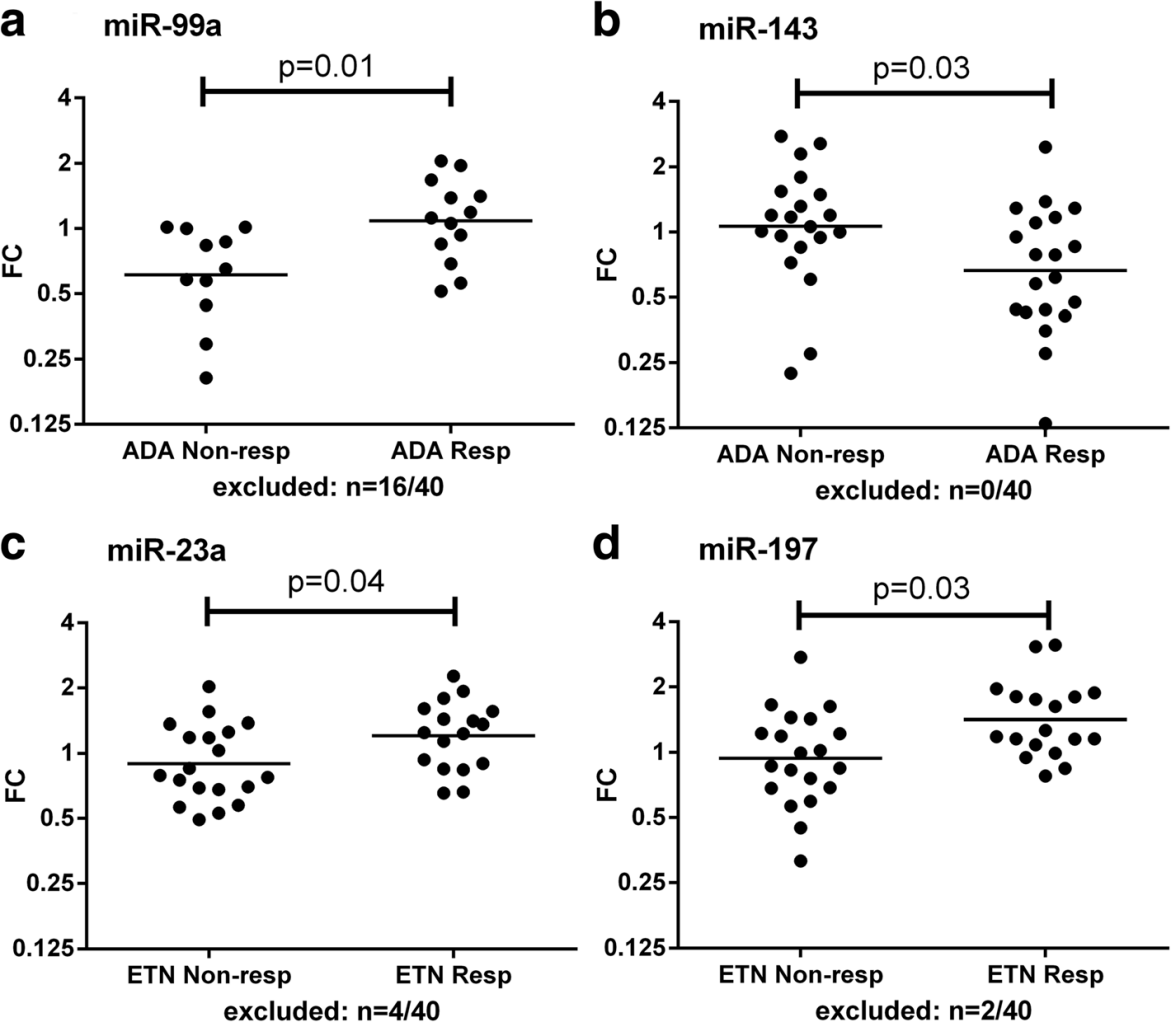
miRNAs significantly differentially expressed in the discovery cohort. A large panel of miRNAs was measured using the OpenArray platform in serum of 40 ADA- and 40 ETN-treated patients. miRNAs showing significant differences (*p* < 0.05) between responders and non-responders were selected as potential predictors. Among all analyzed, four miRNAs were selected as potential predictors. Levels of miRNAs in each individual patient are shown as the fold changes (FCs) for ADA (**a** and **b**) and ETN (**c** and **d**). The geometric mean per group is shown and *p* values between responders and non-responders were calculated on the –ΔΔCrt using an independent sample *t* test. Several patients were excluded from the analysis because of low amplification quality (scores < 1.24): miR-99a (*n* = 16), miR-143 (*n* = 0), miR-23a (*n* = 4), and miR-197 (*n* = 2)

We analyzed the profile of miRNAs present in the circulation of responders versus non-responders with a broad panel of 758 miRNAs. In the discovery cohort (*n* = 80), four miRNAs were significantly differentially expressed between responders and non-responders: high and low baseline levels of respectively miR-99a and miR-143 predicted response to ADA, while patients with high levels of miR-23a and miR-197 more frequently responded to ETN (Fig. 1). Expression values of patients in the discovery cohort were also plotted for miRNAs proposed by the previous studies as predictors for response (Additional file 3). miR-23a in ETN-treated patients was the only miRNA overlapping between this study and previous ones published.

Since the measurement of miRNAs can be costly when incorporated in clinical practice, we wanted to rule out the possibility that the miRNAs identified do not increase the magnitude of prediction that is already possible based on clinical parameters. We therefore compared the predictive abilities of models based on clinical parameters alone, and clinical parameters together with the miRNA expression levels. The clinical characteristics that were used, were those that presented a significant difference between responders and non-responders at baseline, namely the DAS28 (*p* = 0.01), swollen joint count (SJC, *p* = 0.03) and amount of glucocorticoid (GC) users (*p* = 0.01) for patients treated with ADA, and C-reactive protein (CRP, *p* = 0.03) for those treated with ETN (Additional file 1). The predictive properties of these models without and with miRNAs are shown in Table 2. The clinical model for ADA showed a moderate predictive value (AUR-ROC 0.75), that was increased by the addition of miR-99a and miR-143 in the combined model (AUR-ROC 0.97). For ETN, the CRP alone was only limitedly able to predict response (AUC-ROC 0.68), however, the predictive value increased by the addition of miR-23a and miR-197 in the combined model (AUC-ROC 0.78).

**Table 2:** Multivariable models for prediction of response to TNFi

TNFi	Model	Model content	AUC-ROC	Sens.	Spec.
ADA	Clinical	SJC, GC use, DAS28	0.75	80 %	70 %
	Clinical + miRNAs	SJC, GC use, DAS28, miR-99a, miR-143	0.97	92 %	91 %
ETN	Clinical	CRP	0.68	67 %	75 %
	Clinical + miRNAs	CRP, miR-197, miR-23a	0.78	80 %	79 %

Baseline clinical parameters of patients that were different between responders and non-responders (*p* < 0.10) were used to build a “clinical model”. In a “combined model”, the clinical parameters and miRNAs predictive for response were combined, in order to determine the additive value of miRNAs in the prediction of response. For ADA, a model containing the clinical parameters (the square root of) SJC, DAS28 and GC use was compared with a model containing these parameters and the level of circulating miRNAs associated with response to ADA, miR-99a, and miR143 (–ΔΔCrt values). For ETN, the clinical model only contained the (log-transformed) CRP and the combined model also included miR-197 and miR23. Per model AUC-ROC is shown as an indicator of the predictive ability. A useless model would score 0.5, whereas a perfect model would score 1.0. The sensitivity (proportion of positive tests among all responders) and specificity (proportion of all negative tests among all non-responders) were shown for the best cutoff value per model, according to Youden’s index.

*ADA* adalimumab, *AUC-ROC* area under the receiver operating characteristic curve, *CRP* C-reactive protein, *ETN* etanercept, *GC* glucocorticoid, *SJC* swollen joint count, *TNFi* TNF-α-inhibitor

**Fig. 2:**
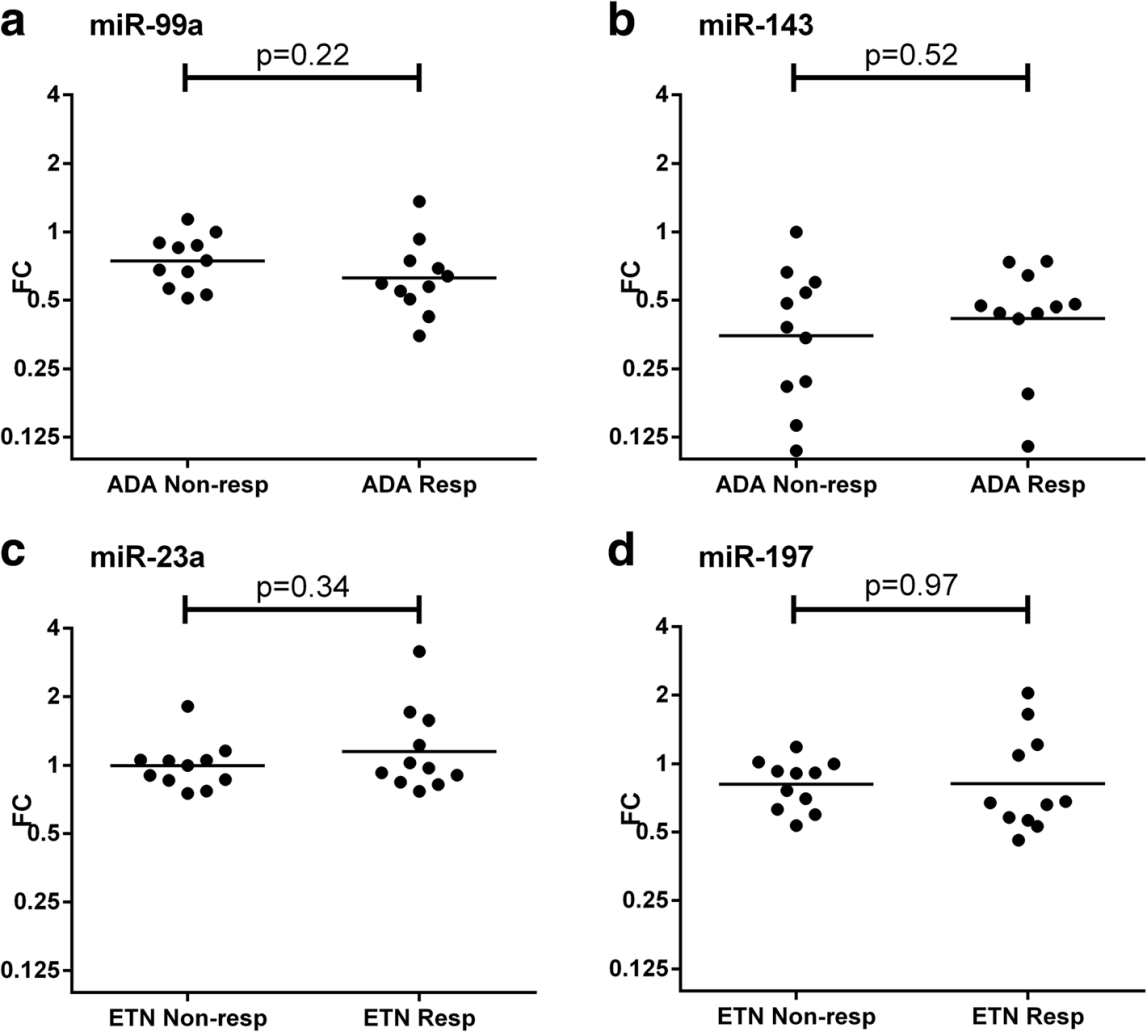
Validation of selected miRNAs. Using single assays, miRNAs selected in the discovery cohort were measured in an independent cohort of patients treated with ADA (**a** and **b**) (*n* = 20) and ETN (**c** and **d**) (*n* = 20). miRNAs were considered validated when showing the “same direction” of variation as in the discovery cohort and a significant difference (*p* < 0.05) between responders and non-responders. Shown are the fold changes (FCs) of the individual patients and the geometric means per group. *P* values were calculated on the –ΔΔCrt using an independent sample *t* test. No patients were excluded from the analysis because of low amplification scores

Since replication in (prognostic) research is key to prove validity, we tried to confirm our results in an additional cohort of 40 patients. The differentially abundant miRNAs from the discovery cohort were analyzed in the validation cohort by using single RT-qPCR assays (Fig. 2). None of the miRNAs could significantly predict the response to TNFi in the validation cohort (*p* > 0.05). For miR-99a and miR-143 in ADA users, inverse directions were seen compared to the results in the discovery cohort.

Multivariable analyses did not confirm the predictive abilities of the combined models found in the discovery phase (Additional file 4). Contrary to what was observed in the discovery phase, application of the prediction model for ADA including only clinical parameters showed better predictive abilities than the corresponding combined model (AUC-ROC from 0.93 to 0.57). This is most likely explained by the inverse relationship of the miRNAs and response in the validation compared to the discovery cohort. For ETN, the addition of miRNA added to the prediction of response (AUC-ROC from 0.59 to 0.66), generating a model that was only limitedly predictive.

### Factors that can contribute to the inability to validate findings in multiple cohorts

It is of importance to investigate which factors are involved in the inability to validate findings in multiple cohorts, since these issues could also be applicable to other studies aiming to identify miRNAs predicting the response to TNFi therapy. Possible (combinations of) factors could be, but are not limited to, the usage of different miRNA detection methods, selection of false positive results in the discovery phase, or clinical parameters influencing the relationship between miRNA levels and response.

Despite that miRNA expression analysis in the discovery and validation phase were both based on the same method of detection, i.e., miRNA-specific retrotranscription combined with TaqMan-based RT-qPCR, the scale (high throughput *versus* single assay) of the techniques sufficiently varies. To evaluate whether these differences could impact the final result, we performed a technical replication in all 40 ADA or 40 ETN samples from the discovery cohort using single assays for the four selected miRNAs (as described in "Methods" - miRNA analyses - Individual miRNA analysis).

**Fig. 3:**
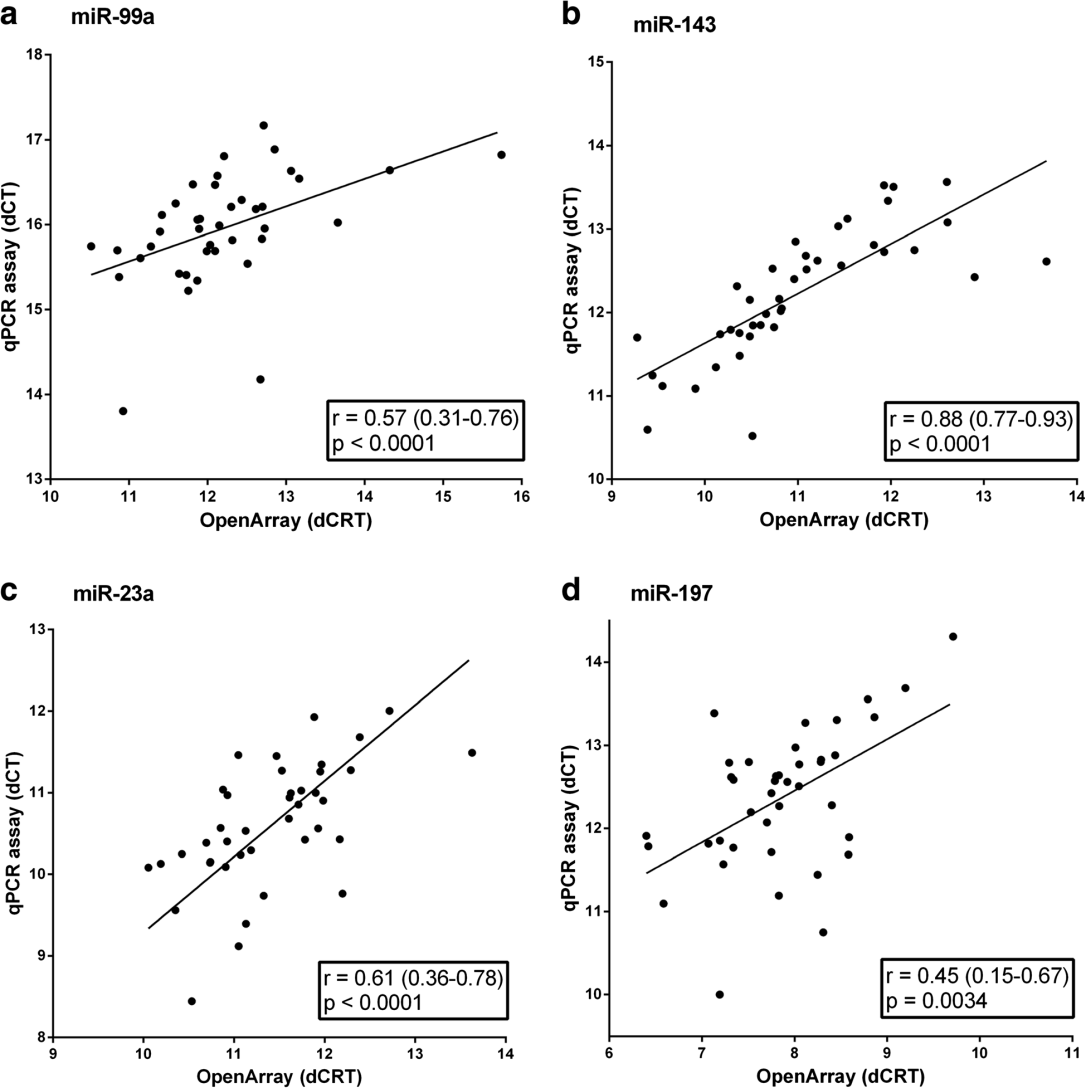
Correlation between OpenArray and single assay results. A technical replication of the four selected miRNAs was performed. Per miRNA, all ADA (**a** and **b**) or ETN (**c** and **d**) samples from the discovery cohort were re-analyzed using TaqMan single miRNA assays. The normalized values for the OpenArray (ΔCrt) and single assay (ΔCt) for all 40 samples was plotted and the Spearman correlation (r) was calculated

Correlations of the results obtained by the profiling *versus* those measured by single assay were assessed by calculating the Spearman correlation (r) between the normalized detection levels (ΔCrt and ΔCt respectively) without excluding samples based on amplification scores (Fig. 3). The correlations of test-retest values ranged from 0.45 to 0.88 (all *p* < 0.0001), which can be considered ranging from reasonable to good, thus demonstrating that the two analyses are concordant. Comparison of the single assay miRNA expression levels between responders *versus* non-responders confirmed that miR-143 was significantly lower in ADA-responders, whereas the other miRNAs showed the same direction as in the profiling, though did not reach significance (Additional file 5).

To verify whether these results could be related to a false discovery rate, we recalculated the differential expression for all miRNAs in the discovery cohort while applying the Benjamini and Hochberg false discovery rate (B&H FDR), which showed corrected *p* values of 1.00 for all miRNAs. Considering none of the miRNAs was significantly different after correction, there is the possibility that only false positive results were selected in the discovery phase.

Another possible explanation why we were unable to replicate the results from previous studies and our discovery cohort is that clinical parameters interact with miRNA levels and these clinical parameters were not equally distributed between the cohorts. Differences in case-mix between cohorts that were unaccounted for (see Additional file 2) would then lead to different estimations of each miRNA and response. Despite this, an adjustment for these clinical parameters would then give comparable estimations for the miRNAs involved. We investigated this theory by running a crude model of response including the specific miRNA only, and an adjusted model considering both the miRNA and clinical parameters, and run these models for the two cohorts analyzed (Table 3). Despite the adjustment, the odds ratios (OR) of these miRNAs for response were still (very) different between the discovery and validation cohort. This indicates that the clinical parameters do not explain why results could not be validated. On the other hand, these analyses showed that clinical parameters have a strong effect on the association between miRNA levels and the response to therapy, as indicated by the (relatively large) differences between crude ORs and adjusted ORs. Therefore, adjustment for clinical parameters will contribute to externalization of results to cohorts with a different case-mix, as is a common occurrence in a heterogeneous disease such as (established) RA. Considering that clinical parameters could affect the expression of miRNAs, miRNA levels may be, to a certain extent, a representation of patient’s clinical characteristics. Therefore, we evaluated the correlation of clinical parameters and miRNAs levels, as measured by single assays, irrespective of response The analysis revealed that all miRNAs associated with either CRP or erythrocyte sedimentation rate (ESR) (Additional file 6). However, since the explained variance of each miRNA by clinical parameters was less than 35 %, miRNA levels are not a complete reflection of clinical characteristics and can thus contain informative additional information.

**Table 3:** Influence of clinical parameters on the association of each miRNA to response

miRNA	Cohort	Crude OR	Crude *p* value	Adjusted OR	Adjusted *p* value
miR-99a	Discovery	6.78	0.03	6.82	0.06
	Validation	0.32	0.28	0.85	0.91
miR-143	Discovery	0.45	0.04	0.39	0.04
	Validation	1.75	0.31	3.43	0.24
miR-23a	Discovery	4.08	0.03	3.82	0.05
	Validation	2.81	0.31	1.86	0.65
miR-197	Discovery	4.32	0.02	5.00	0.03
	Validation	1.41	0.68	0.99	0.99

In order to test if clinical parameters influenced the association between miRNA levels and response, each univariately selected miRNA was first inserted in a logistic regression model on response (crude) in the discovery and validation cohort (–ΔΔCrt and –ΔΔCt values respectively). Then the baseline clinical parameters that were different between discovery and validation (Additional file 2, *p* < 0.10) were added to a separate model (adjusted). The parameters that were used to adjust the association of miR-99a and miR-143 with response to ADA were (log-tranformed) CRP, DAS28, (the square root of) SJC and VAS-GH. The association of miR-23a and miR-197 with response to ETN was adjusted for age and (log-transformed) ESR. If clinical parameters would be the main cause for the inability to validate the findings, the adjusted ORs of the miRNA in discovery and validation should be comparable. The analyses showed that relationship between each miRNA and response was significantly influenced by clinical parameters in most cases; however, because the adjusted ORs between discovery and validation after correction are not comparable, the clinical parameters do not explain the found differences between the different cohorts

*ADA* adalimumab, *CRP* C-reactive protein, DAS28 disease activity score based on a 28-joint count, *ESR* erythrocyte sedimentation rate, *ETN* etanercept, *OR* odds ratio, *SJC* swollen joint count, *VAS-GH* visual analogue scale of general health

Since the exact origin of circulating of miRNAs is unknown and blood cells have been proposed as a key source [27], we wanted to rule out the possibility that the serum levels of miRNAs are a reflection of the composition of circulating leukocytes. We therefore correlated the levels of serum miRNAs with the percentage of peripheral blood leukocyte subsets, as measured by flow cytometry in 20 randomly selected patients (Additional file 7). Out of all comparisons that were considering the surface markers CD3, CD4, CD8, CD14, CD19, CD45, and CD16 + 56 and the FC of all four miRNAs, one significant correlation was observed between the levels of miR-197, as measured in the profiling, and the percentage of natural killer-like T cells (*r* = 0.587, *p* = 0.008). However, this association was not reproduced when considering the miRNA levels measured by the single assay (*r* = 0.083, *p* = 0.831). Even though we cannot exclude the contribution of other rare cell subsets that were not identified, these results suggested that the levels of serum miRNAs in responders *versus* non-responders are not dependent on the composition of circulating leukocytes, thus making a different leukocyte composition an unlikely cause for the inconsistency between the two cohorts analyzed and the other studies.

## Discussion

Prediction of TNFi response is needed for a more personalized approach in RA treatment. Since two previous studies have addressed this question and identified candidate miRNAs [20, 21], we aimed at verifying whether these could be validated in an independent cohort, and eventually, whether we could find new predictors. High values of circulating miR23a were univariately predictive of response in our study and one previous work [20], however, miR-23a was inversely related to response in a third study [21] and is thus not a consistent predictor. On the basis of the profiling results, four miRNAs showed an added value to clinical parameters in predicting response to TNFi. However, these miRNAs could not be validated in a separate cohort of consecutively included patients.

Several factors could have contributed to the inability to replicate findings from previous works and our own selection of miRNAs. A first possible contributing factor is technical variability of the techniques employed for the miRNA analysis. Within our study and the previous studies, pre-analytical and analytical protocols were standardized, as is considered to contribute to more reliable measurements in miRNA studies [28]. Correlations between the two protocols used to analyze miRNA expression were reasonably good, though could explain (some) difference in the outcome. Indeed, a significant differential expression between responders and non-responders could only be replicated for miR-143, which was the miRNA with the best amplification quality in the array among those selected (i.e., amplification score ≥ 1.24 in all samples). The previous studies used comparable techniques for biomarkers discovery: TaqMan single miRNA assays [21] and the same platform as in our discovery step (TaqMan OpenArray, Pool A) [20]. Furthermore, the OpenArray appears one of the most reliable high-throughput technique for miRNA analysis [29], and it was successfully used for profiling of serum miRNA in multiple studies by us [Chouri E, et al, manuscript in preparation] and others [30, 31]. Interestingly, miR-23a was positively related to response both in our discovery cohort and in a previous study that was also using the OpenArray as profiling platform [20]. On the contrary, the absence of a relation or inverse relation with response was found in all cases employing TaqMan single assays [21]. Altogether, the analytical techniques and their technical variability are unlikely to be explanatory for all the differences within and between the studies, though they might be a contributing factor.

The lack of correction for multiple testing in the discovery phase might have led to a subsequent (wrongful) selection of candidate miRNAs. However, a correction for multiple testing was not applied, because this could be too strict when trying to replicate findings already identified by others and would also have increased the chance of excluding potentially useful miRNAs (false negative results). In addition, a separate validation step will reveal which of the (less strictly) selected miRNAs has a true biological meaning, and thus compensates for the absence of correction for false discovery rate. Yet, the fact that the application of B&H FDR resulted in a *p* value of 1.00 for all analyzed miRNAs is suggestive for the possibility that false positive miRNAs were selected. The identification of miRNAs in the previous studies might also be based on false positive results, since no correction for multiple testing was performed and results were not validated in a separate cohort. Not unimportant, if all proposed predictors so far are indeed false positives, this might entail an absence of biologically relevant miRNAs for the prediction of TNFi response in RA.

A third contributing factor for the inability to validate our findings could be related to differences in baseline clinical parameters, which could influence the relation between miRNAs and response. In additional analyses, however, we demonstrated that the clinical parameters were not able to explain why results could not be replicated between the discovery and the validation cohort. However, we did observe that the predictive values of miRNAs were dependent on clinical parameters (large difference in crude OR and adjusted OR) and that, to a certain extent, circulating miRNA levels are a reflection of clinical parameters. Since the other studies did not correct for clinical parameters, heterogeneity in baseline characteristics of included patients might explain why miRNAs were not reproduced across studies. This is especially likely to have occurred if the heterogeneity across studies involves clinical characteristics relating to response, such as baseline DAS28, SJC, GC use and CRP, which were indeed the parameters that differed across the studies considered. In particular, patients included in the work by Castro-Villegas et al. [21] were more frequently treated with GCs (64.7 % *versus* 30.0 %), whereas those in the cohort used by Krintel et al. [20], showed a higher median CRP (15 *versus* 6 mg/l), TJC (15 *versus* 7), SJC (10 *versus* 1) and VAS-GH (70 *versus* 60) as compared to our cohort. In both previous studies the prediction model with miRNAs was not compared to or combined with baseline clinical parameters, which would have made the predictive estimations more generalizable. Concluding, heterogeneity cannot explain why results could not be validated within our study, although it might explain to a certain extent why the predictors identified across the studies are different.

Another possible contributing factor is represented by the chosen study design, in terms of inclusion criteria, measurement and time point of response, statistical analyses, etc. Krintel et al. [20], analyzed a cohort of TNFi-naïve patients treated with MTX and intra-articular triamcinolone, which were additionally randomized to ADA treatment (*n* = 90) or placebo treatment (*n* = 90). To identify miRNAs specifically predictive for EULAR response to ADA combination therapy, an interaction term for each miRNA with the received treatment was added to the prediction model. Castro-Villegas et al. [21] used a cohort of patients treated with ADA, ETN or infliximab and investigated how serum miRNAs changed over time in ten patients after TNFi initiation using miScript miRNA PCR array (Qiagen, Hilden, Germany). In the following step, the ten most relevant miRNAs were measured by single-miRNA assays in 85 additional patients, and univariate and multivariable tests were applied to predict response to any TNFi. In case the true predictive ability of miRNAs is weak, the design might make the difference in inclusion or exclusion of each miRNA, which would explain the differences in identified miRNAs across studies. In addition, discrepancy in found miRNAs across studies might arise from the fact that each TNFi treatment is analyzed independently (our study and Krintel et al. [20]) or in combination with others to find universal miRNAs for TNFi response (Castro-Villegas et al. [21]). Indeed, despite that all registered TNF-alpha- inhibiting therapies target TNF-alpha, they have small chemical differences and etanercept, in particular, also targets lymphotoxin-alpha [32]. It is therefore possible that biomarkers predictive of response to TNFi therapy are to some extent TNFi-specific.

Despite the advantage in terms of stability, the identification of circulating miRNAs with a concrete potential of application in clinical practice is very limited. In other inflammatory diseases, such as inflammatory bowel disease, the usage of miRNAs as potential biomarkers is still being explored, though has so far not revealed usable predictors of response to therapy [33]. In this line, the usefulness and robustness of miRNAs as biomarkers has been questioned; e.g., in non-neoplastic diseases only 33 % (139/416) of the reported miRNAs were considered either biologically plausible, specific for the disease or interpretable with the current knowledge [34]. Another study showed that up to 58 % of the reported circulating miRNAs related to cancer subtypes were not disease-specific and most likely derived from blood cells [35]. These studies indicate that false positive results in studies exploring circulating miRNA are lurking, and warrant additional carefulness when proposing a miRNA as a marker for a specific disease or disease state. For RA specifically, biomarkers are frequently identified in the circulation [13–19]. However, because the disease primarily affects the joints, the synovial compartment might constitute an alternative good source of biomarkers for RA, as has been demonstrated for cell-derived microparticles in the synovium compared to the circulation [36–38]. Since in RA the synovial miRNAs do not necessarily correlate with plasma miRNAs [12], it is possible that synovial miRNAs have better predictive abilities than the circulating ones. If so, incorporation of any predictive test on the synovial fluid or synovial tissue instead of the circulation, will affect the clinical feasibility negatively. In the future, the discovery of potential biomarker can be boosted by the implementation of novel high-throughput techniques. One of the most promising at this regard is next-generation sequencing (NGS) that has the potential to also identify novel and not previously annotated miRNAs (currently 1882 known (http://www.mirbase.org, accessed July 4, 2016 [39]), as it is not restricted to a predefined selection of miRNAs such as the multiplex-based techniques used in this study [40]. NGS might identify previously unknown targets and discover novel miRNAs for the prediction of response to RA.

## Conclusions

So far, there are no miRNAs that can be used in the prediction of response to TNFi therapy. We believe that a combination of differences in study design, technical variability, lack of multiple testing corrections, and heterogeneity between studies could contribute to these discrepancies. However, it is also conceivable that the irreproducibility of results is caused by the absence of truly biologically relevant miRNAs in the prediction of response to TNFi. Overall, our study demonstrated that in order to increase reproducibility of the results, future studies addressing this topic should (1) standardize detection methods, (2) investigate the added value of miRNAs over clinical parameters, (3) technically replicate findings using a method suitable in case of implementation in clinical practice (i.e., single assay), (4) validate findings in a separate cohort, especially when correction for multiple testing in the discovery phase is not performed, and (5) since heterogeneity influences the ORs of miRNAs, prediction models should preferably be validated in a cohort close to the target population.

## Additional files

Additional file 1: Baseline characteristics of responders and non-responders, split for cohort and treatment. (DOCX 22 kb) Additional file 2: Baseline characteristics of patients in the discovery and validation cohort, split for received treatment. (DOCX 19 kb) Additional file 3: Expression of miRNAs predicting TNFi response identified in other studies. In this analysis, the predictive ability of miR-22, miR-23a, miR-223 and miR-886-3p is explored. (DOCX 217 kb) Additional file 4: Validation of multivariable models for prediction of response to TNFi. The prediction models from the discovery cohort were applied in the validation cohort using the miRNA levels as measured by single assays (DOCX 15 kb) Additional file 5: Technical replication of selected miRNAs from the discovery cohort. Using single miRNA assays, the four selected miRNAs were retested in the same patients for their predictive abilities. (DOCX 104 kb) Additional file 6: Clinical parameters at baseline associated with current miRNA levels. Serum values of the four selected miRNAs were tested for their relationship with clinical parameters, independent from response. (DOCX 16 kb) Additional file 7: Cell subsets related to miRNA expression level, as measured by OpenArray or qPCR assay. In this additional analysis, miRNA expression levels are compared with FACS results on PBMC cell subsets. (XLSX 63 kb)

## Abbreviations

ADA: Adalimumab
AUC-ROC: Area under the receiver operating characteristic curve
B&H FDR: Benjamini and Hochberg false discovery rate
BiOCURA: Biologicals and Outcome Compared and predicted Utrecht region in Rheumatoid Arthritis
CRP: C-reactive protein
Crt: relative threshold cycle
Ct: threshold cycles
DAS28: Disease activity score based on a 28-joint count
ESR: Erythrocyte sedimentation rate
ETN: Etanercept
EULAR: European League Against Rheumatism
FC: fold change
GC: glucocorticoid
miRNAs: microRNAs
mRNAs: messenger RNA
NGS: next-generation sequencing
OR: odds ratio
RA: rheumatoid arthritis
RT-qPCR: real-time quantitative PCR
SJC: swollen joint count
TNFi: TNF-α-inhibiting therapy
