# Supplementary material for: Can baseline serum microRNAs predict response to TNF-alpha inhibitors in rheumatoid arthritis?
Source: Arthritis Res Ther. 2016 Aug 24;18(1):189. doi: 10.1186/s13075-016-1085-z (PMC4997731; doi:10.1186/s13075-016-1085-z)
Supplement: Additional file 1: — Baseline characteristics of responders and non-responders, split for cohort and treatment. (DOCX 22 kb) [file 13075_2016_1085_MOESM1_ESM.docx]

**Baseline characteristics of responders and non-responders split for cohort and treatment.** Patients were selected from the observational BiOCURA cohort based on treatment outcome over the course of one year after start of either ADA or ETN treatment. The clinical characteristics reported refer to the values present before treatment initiation. P-values for comparisons between responders and non-responders were calculated by means of an independent sample t-test, Mann-Whitney U test, fisher exact test (2*2) or chi-square (>2*2) based on the distribution of the clinical parameter. Bold p-values indicate significant associations (p<0.05).

|  |  | **Discovery** | | | | | | **Validation** | | | | | |
| --- | --- | --- | --- | --- | --- | --- | --- | --- | --- | --- | --- | --- | --- |
| **Item** | | **ADA (n=40)** | | | **ETN (n=40)** | | | **ADA (n=20)** | | | **ETN (n=20)** | | |
|  |  | **Non-resp. (n=20)** | **Resp. (n=20)** | **p-value** | **Non-resp. (n=20)** | **Resp. (n=20)** | **p-value** | **Non-resp. (n=10)** | **Resp. (n=10)** | **p-value** | **Non-resp. (n=10)** | **Resp. (n=10)** | **p-value** |
| **Female gender, n (%)** | | 15 (75) | 14 (70) | 1.00 | 17 (85) | 15 (75) | 0.70 | 6 (60) | 7 (70) | 1.00 | 8 (80) | 6 (60) | 0.63 |
| **Age, mean years ±sd** | | 53.4 ±11.9 | 56.4 ±12.9 | 0.46 | 56.3 ±9.7 | 53.9 ±10.1 | 0.44 | 56.4 ±8.9 | 47.6 ±10.8 | 0.06 | 62.3 ±6.8 | 57.7 ±11.5 | 0.28 |
| **Current smoker, n (%)** | | 8 (40) | 5 (25) | 0.50 | 7 (35) | 4 (20) | 0.48 | 7 (70) | 4 (40) | 0.37 | 1 (10) | 3 (30) | 0.58 |
| **RF positivity, n (%)** | | 11 (55) | 16 (80) | 0.18 | 14 (70) | 15 (75) | 0.72 | 5 (50) | 5 (50) | 1.00 | 6 (60) | 6 (60) | 1.00 |
| **ACPA positivity, n (%)** | | 12 (60) | 15 (75) | 0.50 | 14 (70) | 17 (85) | 0.13 | 7 (70) | 4 (40) | 0.37 | 5 (50) | 8 (80) | 0.35 |
| **CRP, median (IQR)** | | 10.0 (4.0-13.0) | 7.5 (4.0-12.8) | 0.97 | 4.3 (3.1-7.5) | 8.5 (3.8-30.0) | 0.07 | 1.6 (1.0-6.8) | 2.7 (1.0-10.8) | 0.80 | 2.0 (1.0-23.5) | 8.0 (3.3-12.3) | 0.53 |
| **No. of previously used bDMARDs** | |  |  | 0.14 |  |  | 0.57 |  |  | 0.42 |  |  | 0.55 |
|  | **0, n (%)** | 13 (65) | 17 (85) |  | 15 (75) | 15 (75) |  | 7 (70) | 6 (60) |  | 7 (70) | 7 (70) |  |
|  | **1, n (%)** | 7 (35) | 3 (15) |  | 4 (20) | 5 (25) |  | 2 (20) | 4 (40) |  | 3 (30) | 2 (20) |  |
|  | **2, n (%)** | 0 (0) | 0 (0) |  | 1 (5) | 0 (0) |  | 1 (10) | 0 (0) |  | 0 (0) | 1 (10) |  |
| **Concomitant treatment, n (%)** | | 19 (95) | 19 (95) | 1.00 | 20 (100) | 19 (95) | 1.00 | 10 (100) | 10 (100) | - | 7 (70) | 10 (100) | 0.21 |
|  | **Methotrexate, n (%)** | 13 (65) | 17 (85) | 0.27 | 13 (65) | 16 (80) | 0.48 | 8 (80) | 10 (100) | 0.47 | 5 (50) | 9 (90) | 0.14 |
|  | **Sulfasalazine, n (%)** | 2 (10) | 3 (15) | 1.00 | 3 (15) | 1 (5) | 0.61 | 0 (0) | 1 (10) | 1.00 | 1 (10) | 1 (10) | 1.00 |
|  | **Hydroxychloroquin, n (%)** | 5 (25) | 5 (25) | 1.00 | 7 (35) | 6 (30) | 1.00 | 3 (30) | 2 (20) | 1.00 | 3 (30) | 5 (50) | 0.65 |
|  | **Glucocorticoids, n (%)** | 8 (40) | 3 (15) | 0.16 | 7 (35) | 5 (25) | 0.73 | 6 (60) | 1 (10) | 0.06 | 4 (40) | 1 (10) | 0.30 |
| **Baseline DAS28, mean ±sd** | | 4.3 ±1.3 | 4.8 ±1.0 | 0.15 | 4.4 ±1.2 | 4.8 ±1.0 | 0.28 | 3.2 ±1.3 | 4.5 ±0.7 | **0.02** | 4.1 ±1.2 | 4.5 ±0.8 | 0.55 |
|  | **TJC, median (IQR)** | 7.0 (3.0-13.8) | 7.0 (4.3-14.5) | 0.88 | 4.5 (1.0-21.5) | 6.5 (2.0-17.8) | 1.00 | 2.0 (0.0-11.5) | 5.5 (3.5-14.3) | 0.12 | 7.0 (2.8-15.0) | 4.0 (3.0-10.0) | 0.63 |
|  | **SJC, median (IQR)** | 1.0 (0.0-2.8) | 3.0 (0.0-6.8) | 0.08 | 1.0 (0.0-3.0) | 1.5 (0.3-4.8) | 0.30 | 0.0 (0.0-0.0) | 0.5 (0.0-3.0) | 0.12 | 1.0 (0.0-4.0) | 2.0 (0.8-3.0) | 0.48 |
|  | **VAS, mean ±sd** | 62.0 ±20.7 | 64.5 ±23.8 | 0.73 | 54.5 ±26.0 | 54.5 ±23.9 | 1.00 | 41.5 ±24.7 | 62.5 ±18.7 | 0.05 | 57.5 ±15.9 | 52.0 ±23.0 | 0.54 |
|  | **ESR, median mm/hr (IQR)** | 12.5 (2.5-30.0) | 18.5 (9.0-29.8) | 0.33 | 20.0 (5.3-35.3) | 27.0 (13.0-54.3) | 0.23 | 9.0 (4.5-21.3) | 14.0 (9.3-38.8) | 0.25 | 9.5 (5.3-22.5) | 18.5 (13.0-31.5) | 0.17 |

ACPA: anti-citrullinated protein antibody, ADA: adalimumab, bDMARDs: biological disease modifying antirheumatic drugs, CRP: C-reactive protein, ESR: erythrocyte sedimentation rate, ETN: etanercept, GC: glucocorticoid, HCQ: hydroxychloroquine, IQR: interquartile range, MTX: methotrexate, RF: rheumatoid factor, SJC: swollen joint count, SSZ: sulfasalazine, TJC: tender joint count, VAS-GH: visual analogue scale general health.
