# Supplementary material for: Can baseline serum microRNAs predict response to TNF-alpha inhibitors in rheumatoid arthritis?
Source: Arthritis Res Ther. 2016 Aug 24;18(1):189. doi: 10.1186/s13075-016-1085-z (PMC4997731; doi:10.1186/s13075-016-1085-z)
Supplement: Additional file 3: — Expression of miRNAs predicting TNFi response identified in other studies. In this analysis, the predictive ability of miR-22, miR-23a, miR-223 and miR-886-3p is explored. (DOCX 217 kb) [file 13075_2016_1085_MOESM3_ESM.docx]

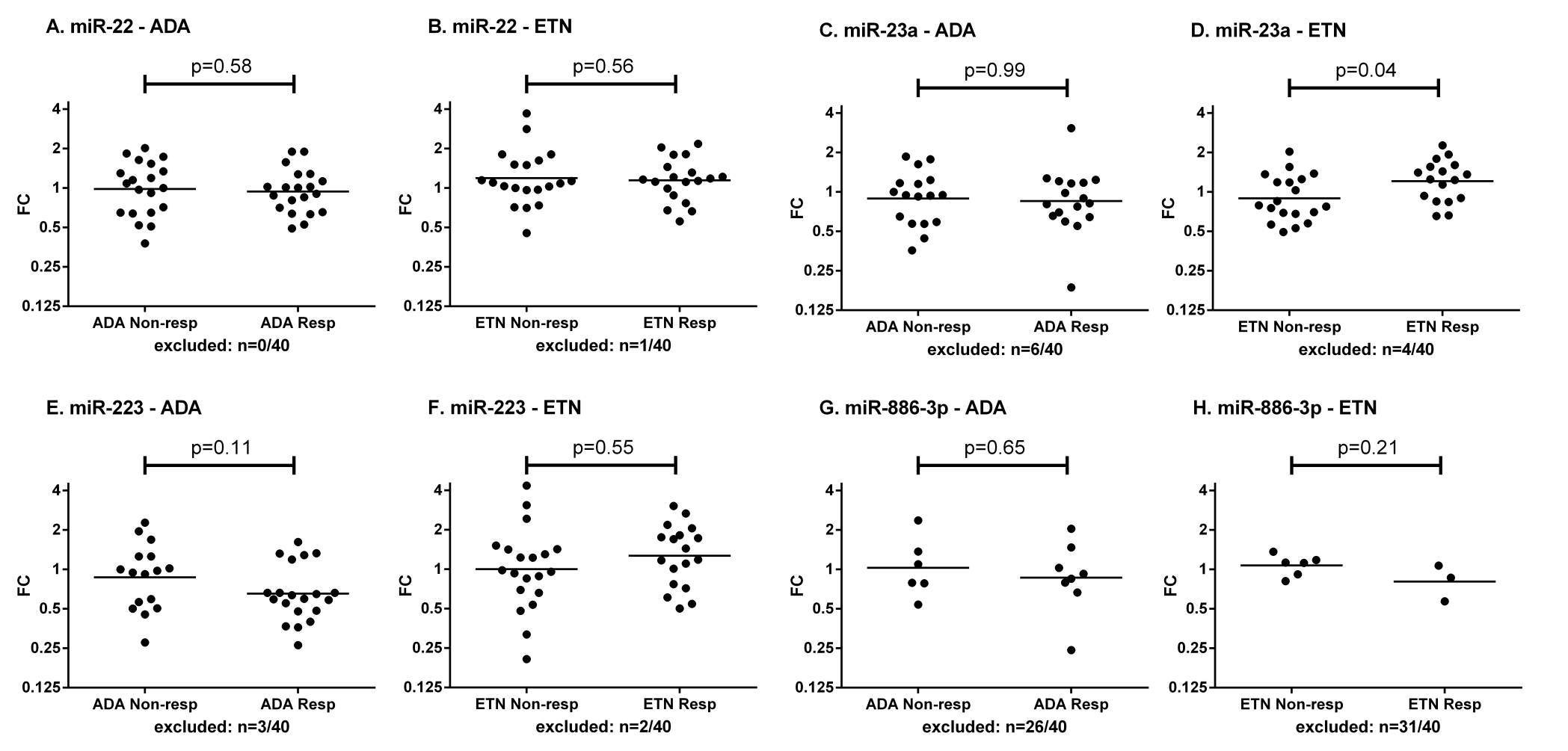
**Expression of miRNAs predicting TNFi response identified in other studies.** In previous studies[20, 21] baseline values of miR-22 (**A** & **B**), miR-23a (**C** & **D**), miR-223 (**E** & **F**) and miR-886-3p (**G** & **H**) were found to be associated with response to TNFi treatment. The expression (FC of individual patients and geometric mean) of these miRNAs are shown in the discovery cohort (n=40 ADA and n=40 ETN) as measured by the OpenArray platform. P-values were calculated on the –ΔΔCrt values using an independent sample t-test.
