# Supplementary material for: Can baseline serum microRNAs predict response to TNF-alpha inhibitors in rheumatoid arthritis?
Source: Arthritis Res Ther. 2016 Aug 24;18(1):189. doi: 10.1186/s13075-016-1085-z (PMC4997731; doi:10.1186/s13075-016-1085-z)
Supplement: Additional file 4: — Validation of multivariable models for prediction of response to TNFi. The prediction models from the discovery cohort were applied in the validation cohort using the miRNA levels as measured by single assays (DOCX 15 kb) [file 13075_2016_1085_MOESM4_ESM.docx]

**Validation of multivariable models for prediction of response to TNFi**. The prediction models from the discovery cohort, based on clinical parameters alone and miRNAs, were applied in the validation cohort (n=20 ADA or n=20 ETN) including the miRNA levels as measured by single assays. For ADA, the clinical baseline characteristics predicting response were (the square root of) the SJC, DAS28, GC use; the combined model included also miR99a and miR197. For ETN, the clinical model contained the (logtransformed) CRP only and the combined model also included miR-23a and miR-143. In order to limit re-fitting, the prediction rules of the clinical and combined models were applied on the validation cohort while freezing the regression coefficients of the individual parameters in the model. Per model, the area under the AUC-ROC was calculated and the sensitivity and specificity were shown for the best cut-off value, according to the Youden’s index.

| TNFi | Model | Model content | AUC-ROC | Sens. | Spec. |
| --- | --- | --- | --- | --- | --- |
| **ADA** | Clinical | SJC, GC use, DAS28 | 0.93 | 90% | 100% |
|  | Clinical + miRNAs | SJC, GC use, DAS28, miR-99a, miR-143 | 0.57 | 50% | 80% |
| **ETN** | Clinical | CRP | 0.59 | 80% | 70% |
|  | Clinical + miRNAs | CRP, miR-23a, miR-197 | 0.66 | 80% | 60% |

ADA: adalimumab, AUC-ROC: area under the receiver operating characteristic curve CRP: C-reactive protein, ETN: etanercept, GC: glucocorticoid, sens: sensitivity, SJC: swollen joint count, spec: specificity, TNFi: TNF-α-inhibitor.
