# Supplementary material for: Can baseline serum microRNAs predict response to TNF-alpha inhibitors in rheumatoid arthritis?
Source: Arthritis Res Ther. 2016 Aug 24;18(1):189. doi: 10.1186/s13075-016-1085-z (PMC4997731; doi:10.1186/s13075-016-1085-z)
Supplement: Additional file 5: — Technical replication of selected miRNAs from the discovery cohort. Using single miRNA assays, the four selected miRNAs were retested in the same patients for their predictive abilities. (DOCX 104 kb) [file 13075_2016_1085_MOESM5_ESM.docx]

**Technical replication of selected miRNAs from the discovery cohort.** Samples from the discovery cohort were re-analyzed using single miRNA assays for the selected miRNAs. If samples were excluded from the discovery step because of low amplification scores, these were also excluded from the technical replication. Graphs for ADA (**A** & **B**) and ETN (**C** & **D**) report the levels of expression as fold change (FC) per individual patient and the geometric means per group (FC). P-values were calculated on the –ΔΔCt values using an independent sample t-test.

**
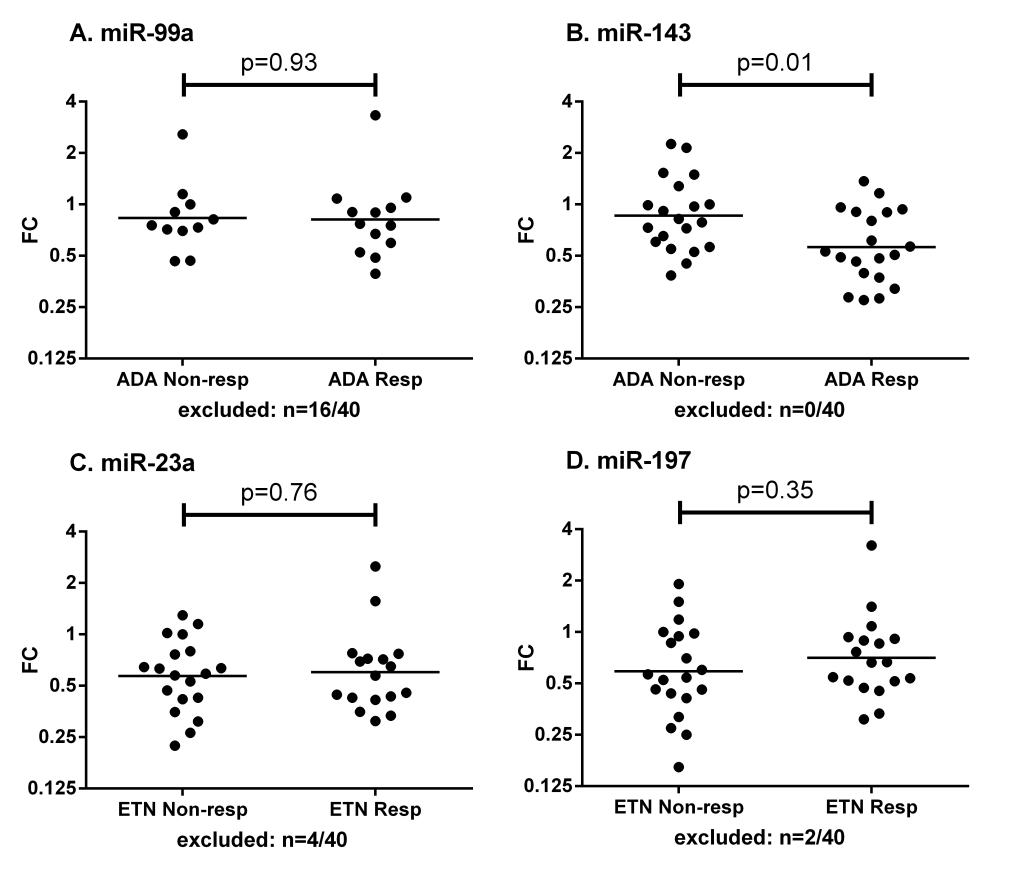
**
