# Supplementary material for: Can baseline serum microRNAs predict response to TNF-alpha inhibitors in rheumatoid arthritis?
Source: Arthritis Res Ther. 2016 Aug 24;18(1):189. doi: 10.1186/s13075-016-1085-z (PMC4997731; doi:10.1186/s13075-016-1085-z)
Supplement: Additional file 6: — Clinical parameters at baseline associated with current miRNA levels. Serum values of the four selected miRNAs were tested for their relationship with clinical parameters, independent from response. (DOCX 16 kb) [file 13075_2016_1085_MOESM6_ESM.docx]

**Clinical parameters at baseline associated with current miRNA levels**. Four miRNAs were measured using Taqman single assays in 80 patient from discovery cohort (i.e. technical replication), and 40 from validation. To evaluate which clinical parameters were associated with current miRNA values, per miRNA a multivariable linear regression model was built with the miRNA level (–ΔΔCt) as dependent variable and baseline clinical parameters as independent variables (full model). Clinical parameters that were entered in the full model were gender, age, smoking status, methotrexate use, hydroxychloroquine use, glucocorticoid use, DAS28, swollen joint count (SJC, square root), erythrocyte sedimentation rate (ESR, natural logarithm) and C-reactive protein (CRP, natural logarithm).Subsequently, a backward selection procedure (p-value>0.05 for exclusion) was used to come to the definitive selection of most influential parameters (final model). Per miRNA, the clinical parameters in the final models were shown that significantly associated with current miRNA levels. The R-square value of each model was determined, as an indication of the explained variance, i.e. the proportion of all variability in miRNA levels that can be explained by the clinical parameters in the model. We did not separate for ADA or ETN treatment received, since all measurements in this analysis were collected before treatment initiation.

| miRNA | **Clinical parameter** | **Effect on miRNA level** | **p-value** | **R-square of model** |
| --- | --- | --- | --- | --- |
| miR99a | CRP | Increase | <0.01 | 0.14 |
| miR143 | Age | Decrease | <0.01 | 0.34 |
|  | CRP | Increase | 0.02 |  |
|  | ESR | Decrease | <0.01 |  |
| miR23a | Gender, female | Decrease | 0.02 | 0.14 |
|  | ESR | Decrease | 0.02 |  |
| miR197 | CRP | Decrease | <0.01 | 0.15 |
|  | ESR | Increase | <0.01 |  |
